# Negativity Bias in Media Multitasking: The Effects of Negative Social Media Messages on Attention to Television News Broadcasts

**DOI:** 10.1371/journal.pone.0153712

**Published:** 2016-05-04

**Authors:** Jari Kätsyri, Teemu Kinnunen, Kenta Kusumoto, Pirkko Oittinen, Niklas Ravaja

**Affiliations:** 1 Department of Computer Science, School of Science, Aalto University, Espoo, Finland; 2 Department of Information and Service Economy, School of Business, Aalto University, Helsinki, Finland; 3 Department of Social Research, University of Helsinki, Helsinki, Finland; 4 Helsinki Institute for Information Technology, Aalto University, Helsinki, Finland; Technion Israel Institute of Technology, ISRAEL

## Abstract

Television viewers’ attention is increasingly more often divided between television and “second screens”, for example when viewing television broadcasts and following their related social media discussion on a tablet computer. The attentional costs of such multitasking may vary depending on the ebb and flow of the social media channel, such as its emotional contents. In the present study, we tested the hypothesis that negative social media messages would draw more attention than similar positive messages. Specifically, news broadcasts were presented in isolation and with simultaneous positive or negative Twitter messages on a tablet to 38 participants in a controlled experiment. Recognition memory, gaze tracking, cardiac responses, and self-reports were used as attentional indices. The presence of any tweets on the tablet decreased attention to the news broadcasts. As expected, negative tweets drew longer viewing times and elicited more attention to themselves than positive tweets. Negative tweets did not, however, decrease attention to the news broadcasts. Taken together, the present results demonstrate a negativity bias exists for social media messages in media multitasking; however, this effect does not amplify the overall detrimental effects of media multitasking.

## Introduction

Television viewers are increasingly more often using secondary media devices such as tablets and mobile phones at the same time as viewing television [1,2]. Although these activities can be unrelated, tablets and mobile phones are also often used as “second screens” to complement the television viewing experience. For example, television viewers can take part in a shared viewing experience by following and commenting upon a social media stream such as Twitter [3] at the same time as viewing the broadcast [4]. Recent theoretical models have highlighted the role of both cognitive capacity limitations and motivational significance on the processing of mediated messages [5,6]. This gives reason to believe that simultaneous social media streams would decrease the allocation of cognitive resources to the television broadcasts, on the one hand, and that these detrimental effects would be amplified when the social media information is highly salient, on the other. In the present study, we investigate whether the affective tone of Twitter messages influences the viewers’ cognitive processing of simultaneously presented television news broadcasts. Our main prediction is that Twitter messages expressing negative attitudes will draw more attention and elicit more elaborate cognitive processing than similar positive Twitter messages.

### Negativity Effects

A unified finding from a diverse field of research ranging from financial decision making to person perception is that negative stimuli elicit greater affective, cognitive, and behavioral influences than equally intense positive stimuli [7–14]. This effect can be explained by the different sensitivities (i.e., activation functions) of the appetitive and aversive motivational systems to positive and negative stimuli, respectively [15]: although appetitive activation is greater in a neutral environment (positivity offset), aversive activation increases more steeply for negative stimuli than appetitive activation does for positive stimuli (negativity bias).

Informational negativity effects, which refer to greater attention to and more detailed cognitive processing of negative than positive stimuli, have been distinguished from affective negativity effects [12,13]. A prime example of an informational negativity effect is that negative words elicit slower performance than positive words in a color-naming task even though the affective tone of words is entirely irrelevant for this task [16]. In the decision making framework, Yechiam and Hochman have theorized that losses (negative events) elicit more on-task attention than equivalent gains (positive events) even when wins and losses do not lead to asymmetries in subjective value [17]. Attention and emotions have also been explicitly considered in the context of mediated message processing. In particular, the Limited Capacity Model of Motivated Mediated Message Processing (“LC4MP”) [5,6] has combined elements from recent psychological theories on both attention and emotion. This model builds upon the assumption that individuals have a limited capacity for cognitive processing of information, and posits that a fixed pool of cognitive resources is divided continuously between information encoding, storage, and retrieval during mediated message processing. For the present purposes, we will focus on the encoding phase, which refers to the selection of information from sensory stores to the working memory. According to LC4MP model, resource allocation to encoding can be indexed by recognition memory performance [5].

The original LC4MP model suggests that increasingly intense appetitive and aversive motivational activations both elicit increased resource allocation to encoding at first but that after an unspecified intensity threshold, aversive activation begins to shift resources away from encoding and towards action preparation [6,18]. Because appetitive activation dominates in the absence of either positive or negative stimuli (i.e., positivity offset), weak positive media messages should be encoded better than weak negative media messages. Intermediate positive and negative messages should receive roughly equal encoding, and the encoding of strong positive messages should again surpass the encoding of strong negative messages. The prediction that negative messages should never receive more encoding resources than positive messages appears to be inconsistent with the known informational negativity effects and the loss attention model of Yechiam and Hochman. Furthermore, it could be speculated that the greater motivational activation elicited by negative than by equally intense positive stimuli (i.e., negativity bias) should also lead to greater allocation of cognitive resources to encoding at least at intermediate stimulus intensities. In fact, in a later development of the LC4MP model, it has been demonstrated that the recognition of moderately negative stimuli does surpass the recognition of moderately positive stimuli [19]. Similarly, some empirical studies have demonstrated better recognition memory for negative than for positive public service announcements [20] and for negative than for positive political advertisements [21].

In the present context, we expected that negative Twitter messages would elicit more attention than equally long and equally intense positive Twitter messages. Attention was indexed by participants’ own estimates, gaze tracking, and recognition memory. News broadcasts and tweets were presented on separate screens so that gaze dwell times (cumulative gaze fixation durations) could be used as a direct measure of visual attention. To summarize, we made the following predictions.

***H1a-c*:** Negative tweets will elicit (a) higher self-reported attention, (b) longer gaze dwell times, and (c) better recognition memory than positive tweets.

The principle of negativity dominance suggests that combinations of positive and negative stimuli should elicit evaluations that are more negative than what would be expected on the basis of the individual negative and positive components [13]. In the present context, the effects of negativity dominance, if existent, would depend on the relative strengths of the news broadcasts and tweets. If news and tweets were roughly equally intense, negative tweets paired with positive news and positive tweets paired with negative tweets should elicit equally strong negative evaluations, and hence no interaction effects between news and tweet valences would follow. On the other hand, if the news were more intense than tweets, negative tweets should shift the evaluation of positive news towards negativity but have lesser or no effects on equally strong negative news. Finally, an opposite pattern could follow if tweets were more intense than news. The following research question was presented for exploring these effects.

***RQ1a-b*:** Will tweet valence interact with news valence when predicting (a) emotional or (b) attentional responses to tweets (as measured by self-reports, recognition memory, and/or gaze dwell time)?

### Media Multitasking

Psychological studies have demonstrated that people are limited in performing two stimulus-response tasks concurrently because cognitive resources required in such tasks can be utilized by only one task at a time (“bottleneck theories”) [22]. Cognitive resources that are critical in this sense include (at least) visual perceptual and declarative memory resources [23]. As discussed above, the LC4MP model [5,6] similarly holds that the attentional processing of mediated messages is limited by the fixed pool of cognitive resources. Both bottleneck theories and the LC4MP model predict that people can process only one visual media stream efficiently at a time.

Previous empirical studies have supported the debilitating effects of media multitasking on cognitive processing. For example, it has been shown that simultaneous text reading impairs recognition memory for television show excerpts [24], that simultaneous listening to an audio podcast impairs recognition and recall memory for an online news story [25], that textual news tickers impair recognition memory for television news broadcasts [26], and that television broadcasts impair recognition memory for textual stimuli [27–29]. Previous studies have typically examined distractor tasks that have been irrelevant for the primary media task. In contrast, the attentional processing of two complementary media tasks could benefit from the semantic similarity between the tasks. For example, a previous study has demonstrated that audiovisual congruity improves the processing of television news broadcasts, plausibly because congruent auditory and visual channels can be processed as a unified semantic unit [30]. Similarly, Wang et al. [31] have postulated that task relevance is one of the main cognitive dimensions of media multitasking. They also demonstrated that relevant media multitasking is preferred over irrelevant media multitasking in daily choices; however, cognitive processing of unrelated and complementary multitasking was not explicitly compared. To our best knowledge, previous studies have not yet demonstrated that complementary media multitasking deteriorates the attentional processing of the primary media. Hence, we posed the following hypothesis.

***H2a-b*:** News presented with tweets will elicit (a) lower self-reported attention and (b) poorer recognition memory than news presented without tweets.

The LC4MP model deposits that orienting responses elicited by stimulus novelty constitute one of the key mechanisms guiding attention during the encoding of mediated messages [5]. Furthermore, the model considers heart rate (HR) deceleration—or, equivalently, cardiac inter-beat intervals (IBI) lengthening—as a reliable index of orienting responses (see also [32]). At first glance, the increased multitasking demands imposed by the simultaneous news broadcast viewing and Twitter feed reading could be expected to elicit a higher frequency of orienting responses and hence longer IBIs (HR deceleration). However, previous studies have demonstrated that moving pictures prompt longer IBIs than static pictures [33,34] and that events in television broadcasts also elicit cardiac decelerations [5,6]. Moving and professionally edited news broadcasts should be expected to trigger a higher frequency of orienting responses than textual Twitter messages. Hence, closer inspection suggests that directing attention away from news broadcasts to Twitter messages should decrease rather than increase orienting responses, which should consequently elicit weaker cardiac deceleration observable as shorter IBIs. That is,

***H2c*:** News presented with tweets will elicit shorter cardiac IBIs than news presented without tweets.

We assumed that if negative tweets would draw more attention to themselves than positive tweets (H1), this increased attention would occur at the cost of attentional processing of news broadcasts. That is, we expected that news broadcasts presented with negative tweets would suffer from more pronounced attentional impairments than news broadcasts presented with positive tweets. Previous media studies without media multitasking have demonstrated greater orienting responses (longer cardiac IBIs) for negative than for positive radio advertisements [35] and for negative than for positive affective images [36]. In the present media multitasking context, we expected that orienting responses would be driven primarily by the television broadcasts (see above). This means that if negative Twitter messages would draw more attention away from the television broadcasts than positive Twitter messages, they should also elicit weaker cardiac orienting responses (shorter IBIs). To summarize, we made the following hypothesis.

***H3a-c*:** News presented with negative tweets will elicit (a) lower self-reported attention, (b) poorer recognition memory, and (c) shorter IBIs than news presented with positive tweets.

A previous study has demonstrated that negative events (losses) in one task can enhance attention to a simultaneously performed secondary task (i.e., an attentional spillover effect) [37]. Although in the present experimental setup it was difficult for the participants to pay attention effectively to both screens (television and tablet) at the same time, we nevertheless cannot fully exclude this possibility. Hence, we note that a plausible alternative hypothesis to H3 is that negative as compared with positive tweets will elicit increased attention to the news broadcasts.

## Materials and Methods

### Participants

Participants were 38 Finnish under- or post-graduate university students (27 male and 11 female; *M* = 25.1 years, *SD* = 4.9 years). All participants were native Finnish speakers, and they reported normal hearing and normal or corrected-to-normal vision. Participants received three movie tickets in compensation for their participation. Gaze tracking data were recorded from a subset of 17 participants (12 male and 5 female; *M* = 25.1 years, *SD* = 2.7 years).

### Ethics Statement

Written consent was obtained from all participants. Our data collection and reporting comply with the Finnish Advisory Board on Research Integrity (TENK) guidelines for human-subject research [38]. Because the present research was non-medical, requirement for prior ethical approval was waived by the Aalto University Research Ethics Committee.

### Design

The experiment used a 2 × 3 × 4 within-subjects design with News Valence (positive, negative), Tweet Condition (positive, negative, control [none]), and Mood (joyful, relaxed, depressed, and fearful) as within-subjects factors. Results for the mood condition, which failed to elicit any significant effects on attention, are available in S1 Appendix. To counterbalance the assignment of news to the 12 conditions (3 tweet × 4 mood conditions), a 12 × 12 Latin square [39] was used. The same Latin square was replicated for the 12 positive and 12 negative news.

### Stimuli

#### News videos

The news stimuli were 24 news video clips. For the preselection, 12 additional participants rated the valence and arousal (both on 9-point scales; see Section Self-report Measures) of 32 news videos selected from regional news broadcasts produced by the Finnish Broadcasting Company [40]. Valence pre-ratings were used to select the 12 most positive (e.g., “Record-breaking salmon summer to be expected on the Tornio River”) and the 12 most negative news videos (e.g., “Hamina struggles with a crippling debt”). Positive and negative news videos received clearly different valence pre-ratings, *M*s = 6.3 and 3.5 (*SD*s = 0.4 and 0.6), *F*(1, 11) = 101.11, *p* < 0.001, *η*^*2*^ = 0.74, but similar arousal pre-ratings, *M*s = 3.7 and 3.8 (*SD*s = 1.4), *F*(1, 11) < 1, *η*^*2*^ = 0.00. The mean video duration for both positive and negative news was 42 s (range 31 to 54 s).

#### Tweets

Four positive and four negative fictional but plausible tweets were created for each news video (in total, 192 tweets). Positivity and negativity were defined as the writer’s attitude towards the topic of the news (e.g., for a debt-related news video, positive message: “A debt crisis is an opportunity for something new” and negative message: “Unbelievable. Keep your debts. I’m moving out”). An initial set of tweets was created by asking each of the 12 pretest participants to write one positive and one negative comment on each news topic. A subset of 192 tweets was then selected and edited, and a panel of three judges classified these tweets as positive or negative. Twelve tweets without a full consensus were rewritten. The average lengths of positive and negative tweets were 67 and 71 characters, *F*(1, 22) = 2.06, *p* = 0.17, *η*^*2*^ = 0.08. Thirteen additional participants were assigned randomly to two groups, which saw separate halves of the tweets, and were then asked to evaluate the attitude of each tweet on a scale ranging from 1 (extremely negative) to 9 (extremely positive). Attitude evaluations for positive and negative tweets were clearly differentiated, *M*s = 7.1 and 2.8 (*SD*s = 0.3 and 0.4), *F*(1, 11) = 627.12, *p* < 0.001, *η*^*2*^ = 0.92. Importantly, positive and negative tweets were also equally intense as measured with distances from the scale middle (i.e., the absolute value of attitude rating minus 5), *M* = 2.4 (*SD* = 0.4) for both.

### Procedure

**Fig 1:**
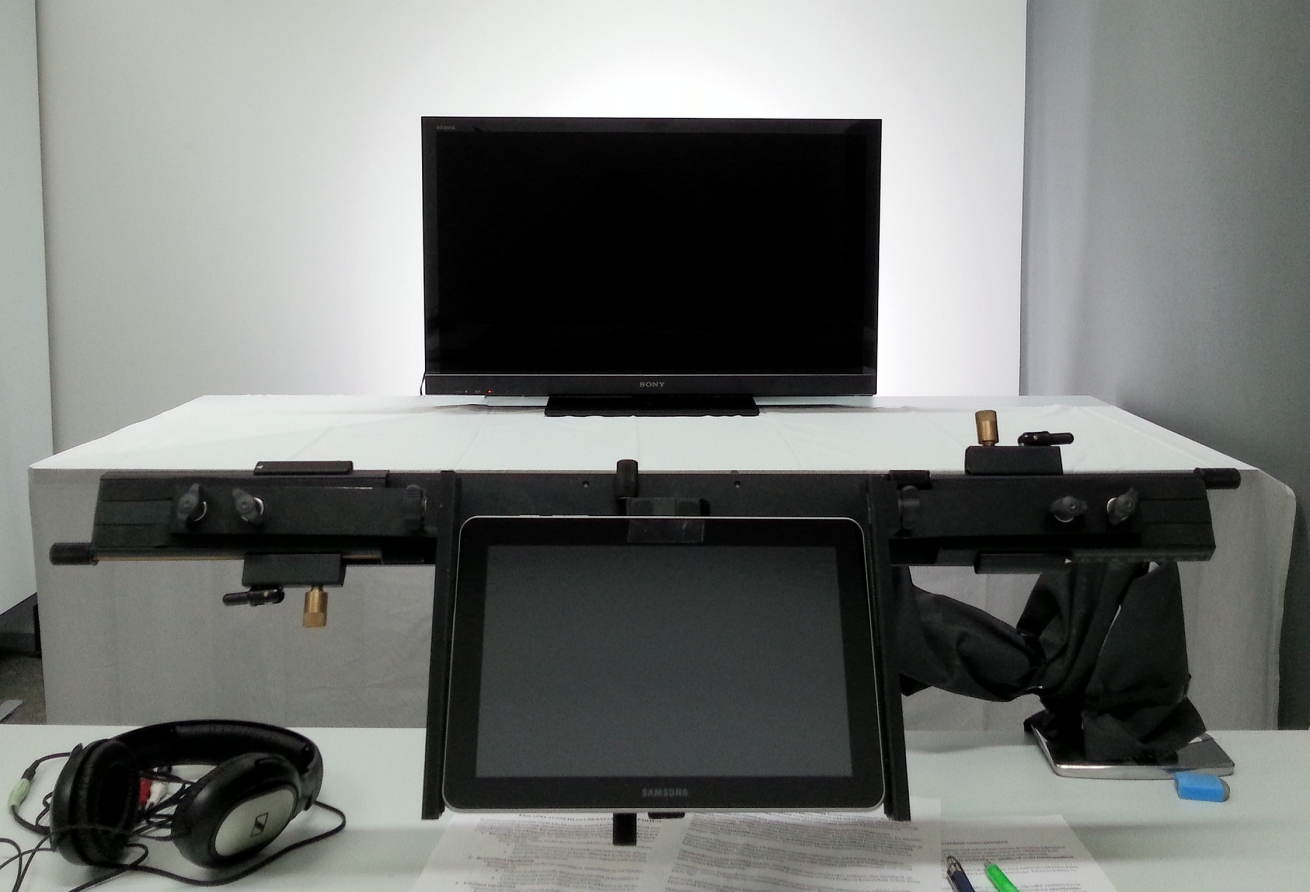
Snapshot of the experimental setup from a participant’s first-person view.

Fig 1 shows the experimental setup from a participant’s perspective. After a brief description of the experiment, the participant filled out background questionnaires. Electrodes were then attached and the participant was seated in a comfortable armchair, followed by a rest period of 5 min for the baseline measurements. Participants were instructed that they would be viewing a number of news videos on the television, some of which would be accompanied by tweets on the tablet, and that they should attempt to memorize the contents of both news and tweets.

Experimental procedure was practiced with one video that was not included in the actual stimuli. During the actual experiment, 24 trials were presented in a randomized order. During each trial, the participant initiated the news video playback by pressing a button on the tablet. The four tweets for each news video, when present, were shown in a random order and at random points of time; however, at least 10 s after the video onset, at most 5 s before the video offset, and with at least 5 s intervals in between. To create a closer resemblance to real Twitter messages, each tweet was presented with a profile image, name, and nickname of its fictional writer. Profile images depicted emotionally neutral faces, user names and nicknames were formed on the basis of common Finnish first and last names, and male and female identities were counterbalanced across messages. When tweets were absent, the tablet remained blank throughout the trial. After each news video, a white fixation cross was presented for 3 s in the center of the television on a medium gray background, after which the participants filled the self-report questionnaires for the trial. After viewing all news videos, the electrodes were removed and the participant completed memory tasks. The participant was then debriefed, and thanked for his or her participation.

News videos were displayed on a 40-inch (88.6 × 49.8 cm or 22.2 × 12.6 degrees of visual angle) Sony Bravia KDL-40HX800 television, which was positioned at a distance of 225 cm from the participant (as per recommendation [41]). News videos were displayed at a spatial resolution of 1024 × 576 pixels and a temporal resolution of 25 frames per second. Sound playback was delivered via closed headphones. Twitter messages were displayed on a 10.1-inch (21.7 × 13.6 cm or 24.5 × 15.5 degrees) Samsung Galaxy Tab tablet positioned on a mount attached to the table in front of the participant, at an approximate distance of 50 cm.

### Self-report Measures

#### Emotional reactions

Participants rated their emotional reactions to news videos (when tweets were not present) or the combinations of news videos and tweets in terms of emotional valence and arousal using 9-point pictorial scales similar to the Self-Assessment Manikin (SAM; [42]). The scales ranged from 1 (very negative or unpleasant) to 9 (very positive or pleasant) for emotional valence, and from 1 (low visceral agitation) to 9 (high visceral agitation) for emotional arousal.

#### Gaze allocation and attention

Participants were asked to give their own estimates of their gaze allocation between the television (news broadcasts) and the tablet (tweets) on a scale ranging from 1 (watched only television) to 4 (watched television and tablet equally) to 7 (watched only tablet). Participants were also asked to evaluate their attention to news and tweets separately using two items: “I attended the news/tweets” and “I was engrossed in the news/tweets” (Cronbachs’ α = 0.84 for both news and tweets). These evaluations were made on a scale ranging from 1 (completely disagree) to 7 (completely agree).

#### Excluded items

Additional media experience self-report items were collected (S1 Appendix) but excluded from the present analysis as irrelevant for the present hypotheses.

### Behavioral Measures

#### Gaze tracking

We used wearable SMI Eye-tracking Glasses (SensoMotoric Instruments GmbH, Teltow, Germany) for gaze position tracking to allow free head movements while switching gaze between the displays. Calibration was done on the television screen using a 3-point calibration procedure provided by the manufacturer, and its success was tested using a custom application that presented a fixation cross sequentially on screen corners in a random order. Due to the limitations of the wearable eye-tracking glasses, gaze tracking data could not be obtained from 7 participants with eye glasses. In addition, calibration test results were unsatisfactory for 14 other participants, which left data from 17 participants for analysis. Gaze data were recorded at 30 Hz temporal resolution. Gaze fixations were detected by the manufacturer’s software, overlain on videos recorded from the participants’ first-person view, and classified manually as falling on television or tablet. Gaze dwell time on the tablet was then calculated for each trial by dividing the cumulative gaze fixation duration on tablet by the sum of the cumulative gaze fixation durations on tablet and television.

#### Recognition Memory

Factual recognition memory for news videos was tested using a knowledge acquisition task (cf. [43,44]). Three questions focusing on central factual details were generated for each news video (e.g., “How large was the debt burden of per resident?”). One correct and three incorrect but plausible response options were given for each question (e.g., 2,500 €; 3,400 €; 4,500 €; and 5,500 €). Recognition memory for tweets was tested by asking each participant to select the four tweet messages that he or she had read for each news video. Twelve options, six positive and six negative, were given for each news video: four target messages with the correct emotional valence (either positive or negative), two foil messages with the same valence, and six foil messages with the opposite valence.

Although the factual recognition memory task has been routinely used to index low-level stimulus encoding [5], we were concerned that the encoding and retrieval of factual items would also depend on higher-level cognitive processing (e.g., associative learning). Consequently, we also included a visual recognition memory task as a more direct index of visual encoding. Visual recognition was tested with a set of 144 images, which consisted of three target images taken from each of the present 24 news videos and three foil images taken from 24 other news videos. Participants saw these images after the actual experiment in a random order and were asked to indicate whether they had seen each image during the experiment. All recognition memory scores were scaled to proportional values between 0 and 1.

### Physiological Measures

Psychophysiological data were recorded with Varioport portable recorder system (Becker Meditec, Karslruhe, Germany) at a resolution of 1 kHz and preprocessed in Matlab (MathWorks Inc., Natick, MA). In addition to cardiac measurements which were used to index attention, we also used facial muscle activation and skin conductance measurements to ensure that our stimuli elicited intended emotional responses in terms of valence and arousal [36], respectively. For each physiological signal, mean values were calculated for each of the full 5-s epochs during news video viewing.

#### Cardiac responses

Electrocardiography signal was recorded using three Ag/AgCl electrodes, pre-filled with hydrogel (model F-55; Skintact, Innsbruck, Austria), and positioned on the chest in a modified Lead III placement. R-peaks were detected from ECG signal using AuBT toolbox [45]. Interbeat-intervals (IBIs) were derived from the R peaks and resampled into equal time intervals every 100 ms. Physiologically unrealistic IBI values (beyond 400–1500 ms, or deviating more than 3 SDs from the participant’s mean) were removed and replaced using cubic spline interpolation. Physiological IBI data from one participant with abnormally high signal variance during the baseline measurement (more than 3 SDs above the mean value of all participants) were removed.

#### Facial muscle responses

Facial electromyography (EMG) activities were recorded from the left *zygomaticus major* (“smiling muscle” on the cheek; ZM), *corrugator supercilii* (“frowning muscle” pulling the eyebrows down diagonally; CS), and *orbicularis oculi* (“crows’ feet wrinkle muscle” orbiting the eye; OO) muscle regions [46] using surface Ag/AgCl electrodes with a contact area of 4 mm diameter (Med Associates Incorporated, St. Albans, VT). Electrodes were filled with electrode gel (model TD-240; Med Assoc. Inc.). Raw EMG data was band-pass filtered (20–500 Hz), band-stop filtered (49–51 Hz), and rectified before mean value extraction. Physiological data from participants with abnormally high signal variance during the baseline measurements (more than 3 SDs above the group’s mean) were removed, resulting in the removal of ZM data from one participant and OO data from another participant. Log transformations were used to reduce positive skew in the EMG data.

#### Skin conductance responses

Electrodermal activity (EDA) signal was recorded by applying a constant voltage of 0.5 V across Ag/AgCl electrodes with a contact area of 4 mm diameter. Electrodes were filled with skin conductance electrode paste (TD-246) and attached to the middle phalanges of the index and little fingers of the participant’s non-dominant hand after hands had been washed with soap and water. EDA signal was downsampled to 10 Hz, smoothed with an adaptive filter, and divided into phasic and tonic components using Ledalab toolbox (version 3.4.4; [47]). Integrated skin conductance responses (iSCRs) were then extracted from the phasic signals as recommended in Benedek and Kaernbach [47]. Physiological iSCR data from one participant with abnormally high signal variance during the baseline measurement (more than 3 SDs above the mean value of all participants) were removed. Log-transformation was used to reduce positive skew in the iSCR data.

### Data Analysis

Because the consecutive physiological measurements were not independent from each other and their number varied depending on the news videos of varying lengths, a conventional variance analysis (ANOVA) would have been inappropriate for the present data. Instead, we opted to use Linear Mixed Model (LMM) analysis, which is an extension of ANOVA analysis that is capable of accounting for both of these problems (for tutorials, see [48–50]). The LMM analysis also allowed us to correctly specify both participants and news stimuli as randomly sampled variables [51]. We used LMM procedure with restricted maximum-likelihood estimation in SPSS (version 22). For specification and explanation of the LMM equations, see S2 Appendix.

All analyses included news valence, tweet condition, and interaction between news valence and tweet condition as fixed factors. Mood condition, which exerted non-significant effects for most variables, was included only for emotional self-reports (SAM valence and arousal) and physiological measurements (facial EMG at ZM, CS, and OO locations; and iSCR). To account for the within-subjects model, random intercepts were always included for participants. Random intercepts for news stimuli (cf. [51]) and random slopes for the fixed factors were also included when they were estimable and at least marginally significant (*p* < 0.20).

For physiological measures, measurement epoch and baseline activity level (both continuous) were included as additional fixed factors, and random slopes for epochs were additionally included to the random part. To account for the non-independence of measurements, epoch was defined as the repeated variable for participants and news videos, and a first-order autoregressive model (AR1) was specified as the error variance-covariance matrix.

Statistical significance level was set to *p* < 0.05 (two-tailed) for all tests. Correction for multiple comparisons was not applied for statistical tests that were planned in advance (i.e., for hypotheses H1 to H3 and RQ1). When applicable, the following planned contrasts were used as follow-up tests for significant effects: “negative > positive tweets”, “no tweets > positive + negative tweets”, and “(negative > positive tweets) × (negative > positive news videos)”.

## Results

### Emotional Responses

Mean values for all dependent variables by news valence and tweet conditions are available in Table 1. Table 2 shows statistical analysis results for emotional response variables. Consistently with our pretest, positive news videos elicited more pleasant SAM valence ratings than negative news videos, 95% CI for the difference [2.19, 2.89] points on the 9-step scale. Consistently with previous facial EMG studies [52], positive news elicited greater ZM and OO activations and weaker CS activations than negative news, 95% CIs [0.03, 0.06], [0.04, 0.09], and [–0.07, –0.12] log(μV) units, respectively.

**Table 1:** Mean results (with SEs) by the interaction between news and tweet valences.

		Negative news			Positive news		
Variable type	Variable	Neg. tweets	Pos. tweets	No tweets	Neg. tweets	Pos. tweets	No tweets
Self-report	SAM Valence	3.78 (0.14)	3.75 (0.14)	3.63 (0.14)	5.88 (0.14)	6.45 (0.14)	6.45 (0.14)
	SAM Arousal	4.45 (0.23)	4.35 (0.23)	4.33 (0.23)	4.00 (0.23)	4.10 (0.23)	4.23 (0.23)
	Gaze on Tablet	3.66 (0.23)	3.53 (0.23)	-	3.82 (0.23)	3.30 (0.23)	-
	Tweet Attention	3.11 (0.23)	2.93 (0.23)	-	3.19 (0.23)	2.82 (0.23)	-
	News Attention	4.15 (0.17)	4.22 (0.17)	4.55 (0.17)	3.83 (0.17)	4.18 (0.17)	4.46 (0.17)
Behavior	Gaze Dwell Time	29% (23%)	28% (23%)	-	33% (23%)	26% (23%)	-
	Tweet Recognition	83% (23%)	77% (23%)	-	82% (23%)	78% (23%)	-
	News Recognition (Factual)	58% (5%)	53% (5%)	59% (5%)	49% (5%)	52% (5%)	63% (5%)
	News Recognition (Visual)	71% (4%)	67% (4%)	78% (4%)	79% (4%)	78% (4%)	88% (4%)
Physiology	EMG-ZM	0.97 (0.03)	0.96 (0.03)	0.96 (0.03)	0.99 (0.03)	0.99 (0.03)	1.03 (0.03)
	EMG-CS	1.62 (0.05)	1.60 (0.05)	1.64 (0.05)	1.52 (0.05)	1.52 (0.05)	1.52 (0.05)
	EMG-OO	0.96 (0.03)	0.95 (0.03)	0.94 (0.03)	1.01 (0.03)	1.01 (0.03)	1.03 (0.03)
	iSCR	0.19 (0.02)	0.20 (0.02)	0.18 (0.02)	0.20 (0.02)	0.18 (0.02)	0.18 (0.02)
	Cardiac IBI	852 (7)	847 (7)	855 (7)	854 (7)	855 (7)	859 (7)

SAM valence and arousal were recorded on a 1–9 scale and other self-ratings on a 1–7 scale. Recognition memory results and tracked gaze allocations on tablet were recorded as proportional values. For SAM valence, higher values denote higher pleasantness. For gaze dwell time, higher values denote more attention on tablet. Physiological measurements were recorded in ln(μV) units for EMG, ln(μS) units for iSCR, and ms units for IBI. SAM = self-assessment manikin; EMG = facial electromyography; ZM = *zygomaticus major* muscle; CS = *corrugator supercilii* muscle; OO = *orbicularis oculi* muscle; IBI = inter-beat interval; iSCR = integrated skin conductance response.

**Table 2:** LMM analysis results for emotional measures.

Variable	Effect	df^a^	*F*	*p*
SAM Valence	News Valence	1, 22^b^	224.05	< 0.001 ***
	Tweet Condition	2, 846	4.95	0.007 **
	News Valence × Tweet Condition	2, 846	9.26	< 0.001 ***
	Mood	3, 844	8.04	< 0.001 ***
SAM Arousal	News Valence	1, 22^b^	1.86	0.187
	Tweet Condition	2, 846	0.16	0.851
	News Valence × Tweet Condition	2, 846	1.32	0.268
	Mood	3, 844	1.29	0.277
Facial EMG-ZM	Baseline	1, 34	43.87	< 0.001 ***
	Epoch	1, 36^c^	28.99	< 0.001 ***
	News Valence	1, 1206	22.62	< 0.001 ***
	Tweet Condition	2, 72^c^	1.05	0.354
	News Valence × Tweet Condition	2, 1207	1.95	0.143
	Mood	3, 108^c^	19.16	< 0.001 ***
Facial EMG-CS	Baseline	1, 36	44.33	< 0.001 ***
	Epoch	1, 37^c^	5.84	0.021 *
	News Valence	1, 1004	56.49	< 0.001 ***
	Tweet Condition	2, 1004	0.75	0.471
	News Valence × Tweet Condition	2, 1005	0.90	0.409
	Mood	3, 111^b^	12.59	< 0.001 ***
Facial EMG-OO	Baseline	1, 35	6.52	0.015 *
	Epoch	1, 37^c^	14.69	< 0.001 ***
	News Valence	1, 36^c^	28.00	< 0.001 ***
	Tweet Condition	2, 1114	0.20	0.822
	News Valence × Tweet Condition	2, 1115	1.72	0.180
	Mood	3, 108^c^	24.80	< 0.001 ***
iSCR	Baseline	1, 31	13.75	< 0.001 ***
	Epoch	1, 34^c^	115.53	< 0.001 ***
	News Valence	1, 1687	0.36	0.551
	Tweet Condition	2, 1688	1.23	0.292
	News Valence × Tweet Condition	2, 1689	0.97	0.378
	Mood	3, 1688	0.96	0.411

SAM = self-assessment manikin; IBI = inter-beat interval; iSCR = integrated skin conductance response; EMG = facial electromyography; ZM = *zygomaticus major* muscle; CS = *corrugator supercilii* muscle; OO = *orbicularis oculi* muscle.

^a^Welch-Sattertwaite approximation (rounded to the closest integer). Note that degrees of freedom for the error term depend on the included random variables.

^b^The model included random intercepts for news stimuli.

^c^The model included random slopes for this term across participants.

*p < 0.05.

**p < 0.01.

***p < 0.001

The main effect of tweet condition on SAM pleasantness ratings was significant; however, this main effect was qualified by a significant interaction between tweet condition and news valence (Table 2). Planned contrast test indicated significant interaction between news and tweet valences (*p* < 0.001). Specifically, participants gave more negative ratings for negative than for positive tweets that were paired with positive news videos (*p* < 0.001), 95% CI [–0.32, –0.82]. In contrast, the effects of negative and positive tweets did not differ when paired with negative news, 95% CI [–0.22, 0.28]. These findings answer our research question RQ1a by demonstrating that negative tweets are emotionally more effective when paired with positive news videos. The effect of negative tweets was much smaller than the effect of negative news (about one tenth; see 95% CIs above). Surprisingly, the main effect of tweet condition and the interaction effect between news valence and tweet condition were both non-significant for all facial EMG responses (Table 2).

No significant effects were observed for SAM arousal ratings (Table 2). In particular, the main effect of tweet condition was non-significant, which suggests that reading tweets simultaneously while watching news did not evoke elevated arousal. Consistently, the effect of tweet condition on physiological arousal (iSCR) was also non-significant (Table 2).

### Attention to Tweets

**Fig 2:**
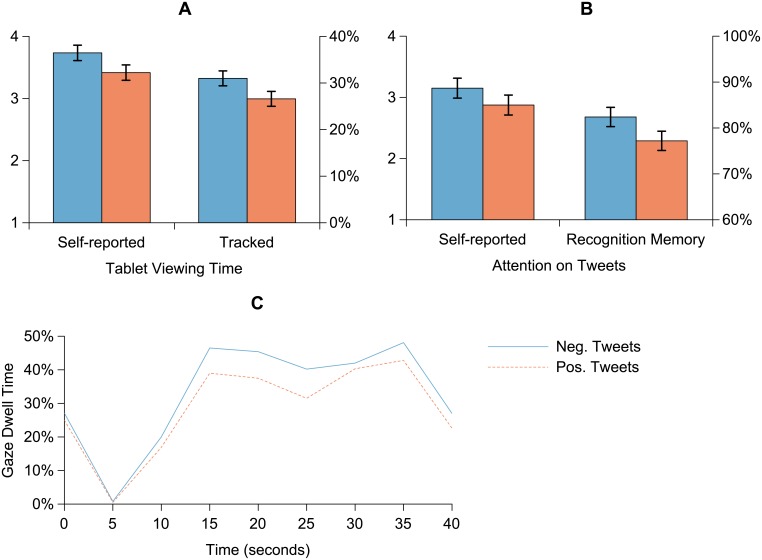
Gaze allocation and attention results for positive and negative tweets. (a) Tweet attention and tablet gaze allocation self-reports. (b) Behavioral attention measure results: tracked gaze allocation on tablet and tweet recognition memory. (c) Average time course for tracked gaze dwell time between television (news broadcasts) and tablet (Twitter messages). The time course has been smoothed with a 5-s moving average filter for illustration (analyses were based on average values). Error bars represent one SEM.

Statistical analysis results for tweet-related attentional measures are shown in Table 3, and tweet-related attentional results for negative and positive tweets are illustrated in Fig 2. Hypothesis H1 predicted that negative tweets would receive more attention than positive tweets. Consistently with H1a, participants’ self-reports indicated significantly greater tweet attention and significantly greater gaze allocation on tablet when negative rather than positive tweets were displayed (Table 3 and Fig 2a), 95% CIs [0.08, 0.47] and [0.06, 0.60] units on the 7-step scale, respectively. Gaze tracking (H1b) and recognition memory task (H1c) provided congruent results: negative tweets elicited significantly longer gaze dwell times on tablet and received significantly better recognition memory than positive tweets (Table 3 and Fig 2b), 95% CIs [2%, 6%] and [2%, 8%], respectively. Fig 2c illustrates the average time course for tracked gaze allocation in more detail. Given that participants initiated a trial by touching the tablet, residual attention on the tablet can still be seen at the very beginning of trials (0 s). After onset (5 s), participants gazed exclusively at the television. Once the presentation of Twitter messages had begun (10 s onwards), participants began to divide their attention between the tablet and the television with an overall preference for the latter. As can be seen, gaze dwell times on tablet were at this phase consistently longer for negative than for positive tweets.

**Table 3:** LMM analysis results for tweet-related attentional measures.

Variable	Effect	df^a^	*F*	*p*
SR Tweet Attention	News Valence	1, 21^b^	0.01	0.936
	Tweet Condition	1, 37^c^	8.18	0.007 **
	News Valence × Tweet Condition	1, 514	1.62	0.204
SR Gaze on Tablet	News Valence	1, 21^b^	0.06	0.809
	Tweet Condition	1, 36^c^	6.07	0.019 *
	News Valence × Tweet Condition	1, 516	4.38	0.037 *
Tracked Gaze on Tablet	News Valence	1, 22^b^	0.12	0.728
	Tweet Condition	1, 231	16.17	< 0.001 ***
	News Valence × Tweet Condition	1, 231	6.15	0.014 *
Tweet Recognition	News Valence	1, 22^b^	0.00	0.988
	Tweet Condition	1, 552	13.27	< 0.001 ***
	News Valence × Tweet Condition	1, 552	0.63	0.429

SR = self-reported.

^a^Welch-Sattertwaite approximation (rounded to the closest integer). Note that degrees of freedom for the error term depend on the included random variables.

^b^The model included random intercepts for news stimuli.

^c^The model included random slopes for this term across participants.

*p < 0.05.

**p < 0.01.

***p < 0.001

RQ1b asked whether attentional responses to tweets would depend on the interaction between news and tweet valences. Interaction between news valence and tweet condition was significant for both self-reported and tracked viewing time measures (Table 3). As can be seen in Table 1, both of these variables showed a similar interaction effect. For positive news videos, negative tweets elicited significantly longer viewing times than positive tweets, 95% CIs [0.20, 0.85] and [4%, 10%] for self-reports and gaze dwell times, respectively. For negative news videos, the effects of negative and positive tweets did not differ, 95% CIs [–0.19, 0.45] and [–1%, 5%]. The interaction effect between news valence and tweet condition was not significant for self-reported attention on tweets or for tweet recognition memory (Table 3).

### Attention to News Videos

**Fig 3:**
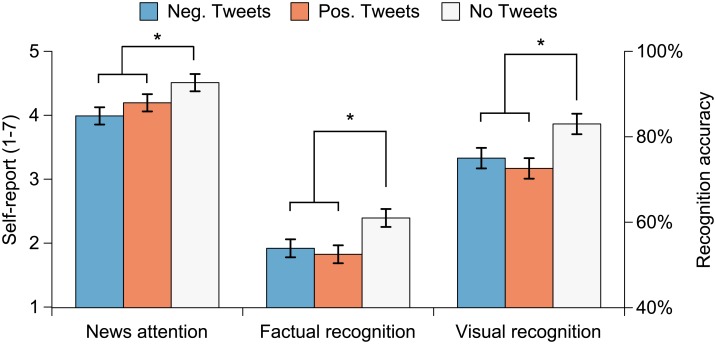
News attention self-report and recognition memory results by tweet condition. Significant differences between conditions are marked with an asterisk (‘*’). Error bars represent one SEM.

In hypothesis H2 we predicted that the presence of any tweets would decrease attention to news videos. Table 4 shows statistical analysis results for news-related attentional variables, and Fig 3 illustrates attentional self-report and recognition memory results for news by tweet condition. The main effect of tweet condition was significant for self-reported news attention and both news recognition memory variables (Table 4). As expected (H2a), news presented with tweets received significantly lower self-reported attention than news presented without tweets (*p* < 0.001), 95% CI for the difference [–0.22, –0.61] points on the 7-step scale. Similarly (H2b), news presented with tweets elicited significantly poorer factual (*p* < 0.001) and visual recognition memory (*p* < 0.001), 95% CIs [–3%, –12%] and [–6%, –12%].

**Table 4:** LMM analysis results for news-related attentional measures.

Variable	Effect	df^a^	*F*	*p*
SR News Attention	News Valence	1, 22^b^	1.36	0.257
	Tweet Condition	2, 73^c^	10.34	< 0.001 ***
	News Valence × Tweet Condition	2, 775	1.57	0.209
News Recognition (Fact.)	News Valence	1, 22^b^	0.20	0.659
	Tweet Condition	2, 73^c^	6.02	0.004 **
	News Valence × Tweet Condition	2, 776	4.55	0.011 *
News Recognition (Vis.)	News Valence	1, 22^b^	4.58	0.044 *
	Tweet Condition	2, 848	17.01	< 0.001 ***
	News Valence × Tweet Condition	2, 848	0.67	0.514
Cardiac IBI	Baseline	1, 35	321.32	< 0.001 ***
	Epoch	1, 37^c^	117.25	< 0.001 ***
	News Valence	1, 26^b^,^c^	1.48	0.235
	Tweet Condition	2, 71^c^	2.95	0.059
	News Valence × Tweet Condition	2, 1457	0.99	0.372

SR = self-reported.

^a^Welch-Sattertwaite approximation (rounded to the closest integer). Note that degrees of freedom for the error term depend on the included random variables.

^b^The model included random intercepts for news stimuli.

^c^The model included random slopes for this term across participants.

*p < 0.05.

**p < 0.01.

***p < 0.001

**Fig 4:**
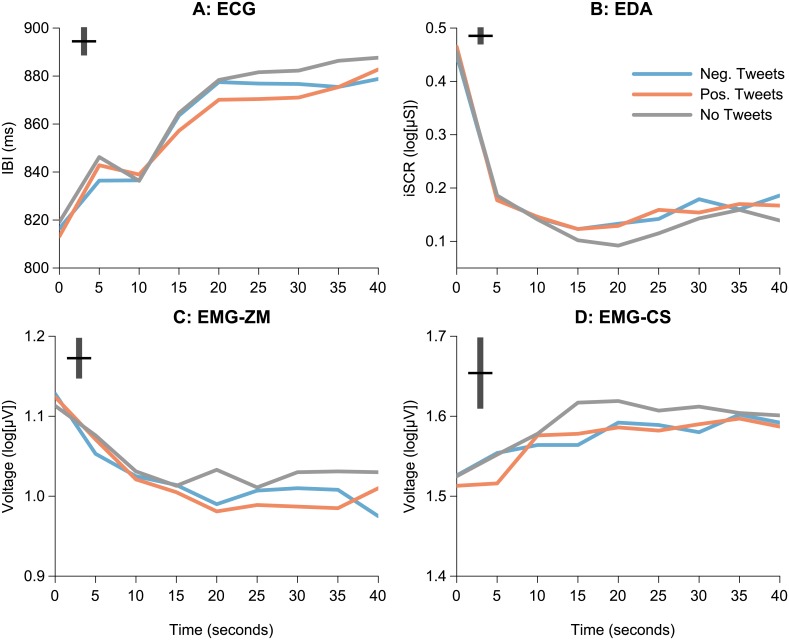
Psychophysiological activations by tweet condition. Average time courses for (a) cardiac responses (IBI), (b) skin conductance responses (iSCR), (c) facial electromyography responses at zygomaticus major location (EMG-ZM/OO), and (d) facial electromyography responses at corrugator supercilii (CS) location. Facial EMG activations at orbicularis oculi (OO) location were almost identical to those of EMG-ZM and are not presented separately. Time courses are presented for 5-s epochs. Black bars at the upper corners represent ± 1 SEM.

Hypothesis H2c predicted that news presented with tweets would elicit shorter IBIs (i.e., weaker cardiac orienting responses) than news presented without tweets. Fig 4 illustrates average time courses within a trial for all physiological variables by tweet condition. As can be seen in Fig 4a, cardiac IBIs showed an overall increasing trend (cardiac deceleration) in all conditions. A slight decrease (cardiac acceleration) can be seen 10–15 s after the trial onsets, which apparently reflects a late cardiac response to heightened sympathetic arousal at the beginning of trials (cf. iSCR responses in Fig 4b). Consistently with H2c, both positive and negative tweets appeared to elicit shorter IBIs beginning approximately 25 s after the trial onsets. Although the main effect of tweet condition on IBI responses was only marginally significant (*p* = 0.059; Table 4), planned contrast for positive and negative versus no tweets reached statistical significance (*p* = 0.028). Consistently with our prediction (H2c), news presented with tweets elicited significantly shorter average IBIs than news presented without tweets, 95% CI [–1, –10] ms.

We also predicted that negative tweets would draw more attention away from the news videos than positive tweets (H3). Although the main effect of tweet condition was significant for self-reported news attention and both recognition memory scores (Table 4), planned comparisons did not indicate significant differences between negative and positive tweets. News videos presented with negative tweets received marginally but not significantly (*p* = 0.073) lower news attention ratings than news videos presented with positive tweets (H3a), 95% CI [–0.44, 0.02]. Contrary to H3b, news videos presented with negative tweets did not receive significantly poorer factual (*p* = 0.693) or visual recognition scores (*p* = 0.186) than news videos presented with positive tweets, 95% CIs [–4%, 6%] and [–1%, 6%], respectively. Furthermore, cardiac IBIs did not differ significantly for news presented with negative and positive tweets (H3c; *p* = 0.356), 95% CI [–8, 3] ms.

Table 4 also shows two unpredicted statistically significant effects. First, the main effect of news valence on visual recognition scores was significant such that positive news were recognized better than negative news, 95% CI [0%, 20%]. Second, the interaction between news valence and tweet condition was significant for factual recognition scores. A follow-up test for the interaction between news and tweet valences was not significant (*p* = 0.084), however. Specifically, the effects of negative and positive tweets did not differ significantly when paired with negative and positive news videos, 95% CIs [–9%, 4%] and [–2%, 12%], respectively. Strictly speaking, statistical significance levels in these unplanned tests should have been corrected for multiple comparisons. Given that these effects would not have survived such correction (corrected α = 0.05/12 = 0.004 [for 12 dependent variables]) and neither one of them was significant for both recognition scores, we considered these effects as chance findings.

## Discussion

The present investigation studied the attentional effects of negative and positive Twitter messages on simultaneously presented news broadcasts. The main finding was that negative tweets draw more gaze dwell time and are recognized better than positive tweets. Increased gaze dwell time on negative tweets was supported both by gaze tracking data and subjective evaluations. These results cannot be explained by varying Twitter message lengths or intensities, given that the positive and negative tweets were matched in length and the strength of their expressed attitudes. Furthermore, tweet conditions were counterbalanced within the individual news, which means that news-specific idiosyncratic results should have been averaged out.

The above results are consistent with well-known informational negativity effects, which have demonstrated that negative stimuli prompt more attention and more detailed cognitive processing than equally intense positive stimuli [12,13]. Participants’ self-reports and tracked gaze dwell times can be considered as direct subjective and objective attentional indices, respectively. Furthermore, recognition memory has been considered as a good index of mediated message encoding [5]. Hence, the present better recognition memory results would suggest that more resources were allocated to the encoding of negative tweets than to the encoding of positive tweets. Better recognition memory results for negative tweets are consistent with previous studies which have, for example, demonstrated enhanced recognition memory for negative than for positive public service announcements [20] and political advertisements [21].

Our results confirmed that the presence of tweets—that is, media multitasking—decreases attention to news as measured with both self-reports and recognition memory. This finding is consistent with the LC4MP model [6], which suggests that limited resources need to be allocated simultaneously to encoding, storage, and retrieval processes. Given that textual tweets were presented simultaneously with a continuous audiovisual news stream, encoding resources assigned to tweets should have reduced similar resources for news. Other previous studies have demonstrated that a simultaneous reading task elicits decreased recognition memory for television entertainment broadcasts [24] and that textual news tickers elicit decreased recognition memory for news broadcasts [26]. Our recognition memory results are consistent with these findings. Furthermore, the present results demonstrate that semantic similarity between the secondary and primary media tasks does not compensate for the increased attentional cost of media multitasking. The extent of semantic similarity between the present social media messages and news broadcasts could be debated, however, because the former addressed viewers’ opinions about the news rather than provided additional information about the news themselves. It is possible that the disadvantages of media multitasking could vary depending on whether the two media channels are fully complementary (e.g., news broadcasts and textual messages repeating their focal contents), related (e.g., the present task) or unrelated (e.g., news broadcasts and unrelated twitter messages). This question could be investigated in future studies by explicitly comparing such conditions with each other.

A limitation of the present findings is that participants were explicitly required to attend both news and tweets, which differs from a natural viewing condition in which viewers can choose for themselves on what to focus and when. Consequently, it is possible that the detrimental effects of multitasking could be weaker in everyday media use. Another limitation of the present study is that our measurements tapped only into the encoding phase of the LC4MP model [5], whereas tweets could have been expected to influence also the storage and retrieval of information. Storage and retrieval performance could be tested in future studies, for example, by utilizing cued and free recall memory tasks. Secondary task reaction time measurements could also be used as an additional index of encoding [5].

We expected that because cardiac decelerations indicative of orienting responses should be driven mainly by events in the television broadcasts, the presence of tweets should decrease cardiac decelerations (i.e., elicit relatively shorter IBIs). Our results gave tentative evidence for this prediction even though the results failed to reach statistical significance and the effect size was small (i.e., at most 10 ms shorter IBIs for news presented with tweets). However, we note that the straightforward IBI measure is limited as an attentional index because it is reciprocally sensitive to both arousal and attention [32]. Heart rate variability measures such as the respiratory sinus arrhythmia (RSA) would have been more unequivocal attentional measures, however, this option was not available for the present analyses given that frequency domain calculations for RSA would have required longer stimuli (60 s at the bare minimum [53]). Furthermore, it is unlikely that heightened arousal would have confounded our IBI results given that the presence of tweets did not elicit elevated arousal as measured with EDA activity or SAM arousal self-reports. That is, given that EDA is unilaterally sensitive to sympathetic arousal [32], it is plausible that the observed small IBI changes were due to attentional rather than arousal effects.

We also predicted that the increased attention to negative tweets would occur at the cost of decreased attention to news broadcast. Our results did not support this prediction, however. Self-reported news attention was marginally lower for news presented with than without tweets; however, this findings was not corroborated by recognition memory results. Taken together, the present results suggest that although negative social media messages draw more attention towards themselves, participants are able to compensate for the imposed attentional demands. A limitation of the present study is that we used a fixed and relatively low frequency of twitter messages. Increasing the number of tweets per each news broadcast could increase the attentional demands of tweets and also compromise the cognitive processing of news. Hence, future studies could test whether increasing the number of tweets from that of the present study would elicit more pronounced attentional effects for tweets in general and negative tweets in particular.

Although tweets expressing other individuals’ negative and positive attitudes towards the news broadcasts modulated participants’ retrospective pleasantness evaluations, facial EMG measurements failed to indicate consistent emotional reactions at the time when the news and tweets were being viewed. This was somewhat unexpected, given that facial EMG measurements are recognized as a valid index of emotional valence [36] and they were in the present investigation clearly sensitive to the valence of news and the valence of background mood (as described in S1 Appendix). The present findings suggest that negative and positive social media messages modulate retrospective judgments but that they do not function as emotional signals *per se*. This suggestion is consistent with the affect heuristic framework [54], which suggests that objects are tagged to varying degrees with positive or negative affective tags that are used as heuristic cues when making evaluative judgments. In the present study, positive and negative tags may have become associated with news broadcasts while they were being viewed and consequently functioned as affective tags in retrospective judgments.

The principle of negativity dominance [13] led to the research question of whether the emotional and attentional effects of negative tweets would be stronger when paired with positive than with negative news. We found that negative as compared with positive tweets elicited greater self-reported unpleasantness but only when they were paired with positive news. Similar effect was observed for self-reported and tracked gaze allocation measures. Furthermore, our results showed that television was clearly the primary media in the present context. Hence, consistently with the principle of negativity dominance, the present results indicate that even weak negative signals (tweets) can modulate emotional and attentional responses to strong positive stimuli (news), whereas weak positive signals do not exert similar effects on strong negative signals. Similarly as above, however, negative tweets did not compromise the attentional processing of news information even when they were paired with positive news broadcasts.

Taken together, our results demonstrate for the first time that negative information presented on a second screen draws more attention to itself than similar positive information. This finding is a novel replication of the negativity bias phenomenon in the context of media multitasking. However, although negative information drew more attention to itself, this effect was not sufficiently strong to compromise the attentional processing of the primary media. On the other hand, our results do demonstrate that second-screen information has significant debilitating effects on attention. These results have practical implications for the television industry. Many television shows have deliberately incorporated Twitter feedback in their broadcasts. Although this parallel tweet narrative may enrich the viewing experience, it also inevitably draws attention away from the primary broadcast. Television broadcast producers might hence want to avoid presenting second-screen information in cognitively demanding parts of their programs. Although the present study demonstrates that negative emotional information presented on a second screen draws more attention than equally strong positive information, the present findings also suggest that this effect does not inflate the detrimental effects of any information presented on second screens.

## Supporting Information

S1 Appendix Excluded data. (PDF)

S2 Appendix Linear Mixed Model equations. (PDF)

S3 Appendix In accordance with the guidelines of PLOS ONE concerning data availability, Appendix S3 contains all SPSS data analysis files for the experiment. To protect participants’ privacy, participant identifiers have been anonymized and no participant-related data are included in the data files.
(ZIP)
