# Supplementary material for: Negativity Bias in Media Multitasking: The Effects of Negative Social Media Messages on Attention to Television News Broadcasts
Source: PLoS One. 2016 May 4;11(5):e0153712. doi: 10.1371/journal.pone.0153712 (PMC4856346; doi:10.1371/journal.pone.0153712)
Supplement: S1 Appendix — (PDF) [file pone.0153712.s001.pdf]

S1 Appendix: Excluded Data

1 Excluded Data

1.1 Mood Condition

Mood induction was originally included in the experimental design as an additional reference for the attentional effects of tweets. The inclusion of mood was motivated by a line of research evidence demonstrating that negative mood fosters a narrow attentional focus on central details, whereas positive mood elicits a broader attentional scope [1–3]. In the present context, tablet screen constituted a more peripheral information source than the television. Based on these findings, we expected that negative as compared with positive mood should draw the viewers' attention towards television screen (news videos) at the cost of the tablet (Twitter messages). To test this prediction, we used a similar autobiographical mood induction procedure as Ravaja et al. [4]. At the beginning of experiment, the participant was asked to write four short notes describing events that had evoked joy, relaxation, fear, and depression the most powerfully in his or her life. Each experimental trial begun with a 15-s mood induction phase, during which the participant was asked to create a vivid mental image of reliving the events pertaining to the specific mood condition. Participants were explicitly told to not show the notes to the experimenter in any phase of the experiment. Participants were asked to memorize the written notes and to dispose them after finishing the experiment. Mood induction procedure was practiced both with and without the notes prior to the experiment.

1.2 Media Experience Measures

We used the following self-report evalutions to gain a comprehensive view of the viewers' media experiences [5] elicited by the news videos and tweets: news and tweet liking, news and tweet involvement (two items similar to [4]: "I found the news/tweets personally relevant" and "I found the news/tweets interesting"; $\alpha = 0.82$ for news and 0.90 for tweets), tweet reading effort, news comprehension, news newsworthiness, news trustworthiness, and news consumption intent (three items similar to [6]: "I would pay for this news message", "How much would you pay for this news message?", "I would share this news message with a friend"; $\alpha = 0.86$). All items were evaluated on a scale ranging from 1 (totally disagree / nothing) to 7 (totally agree / very much).

2 Results and Discussion

2.1 Mood Condition

Mood effects were analyzed using similar LMM analyses as in the main article (see section Data Analysis) except for the inclusion of mood as an additional fixed effect. Statistical analysis results for the mood condition are shown in Table S1.1. As can be seen, mood exerted significant effects only on emotional valence related variables. Mean values for these variables by mood condition are shown in Table S1.2. The results demonstrate that joyful mood elicited higher pleasantness ratings and both more positive (increased EMG-ZM and EMG-OO activations) and less negative (decreased EMG-CS activations) facial EMG responses than relaxed, fearful, or depressed mood. Relaxed mood elicited less negative facial EMG responses (decreased EMG-CS activations) but not consistently more positive facial EMG responses (increased EMG-ZM or EMG-OO activations) than fear and depression. These findings are qualitatively similar to those reported previously by Ravaja et al. [4], who showed that *a priori* positive mood (joy and relaxation) elicits more positive and

less negative emotional reactions *a priori* negative mood (fear and depression). However, the present findings extend those of Ravaja et al. by demonstrating a more fine-grained distinction between joy and relaxation. That is, whereas joyful mood elicited clear emotional effects both in terms of positive and negative valence (pleasantness and unpleasantness), the effects of relaxed mood were restricted to negative valence. Given that mood did not elicit any significant effects on tweet attention (Table S.1.1), the results failed to support our prediction that negative mood would decrease attention to tweets.

Table S1.1. LMM analysis results for emotional and attentional variables by mood.

Variable Type	Variable	df[a]	*F*	*p*	
Emotion SR	SAM Valence	3, 844	8.04	< 0.001	***
	SAM Arousal	3, 844	1.29	0.277	
Emotion PHY	EMG-ZM	3, 108[b]	19.08	< 0.001	***
	EMG-CS	3, 111[b]	12.59	< 0.001	***
	EMG-OO	3, 108[b]	24.80	< 0.001	***
	iSCR	3, 1688	0.96	0.411	
Attention SR	Tweet Attention	3, 507	0.71	0.548	
	Gaze on Tablet	3, 509	1.75	0.156	
	News Attention	3, 769	0.17	0.917	
Attention BEH	Tracked Gaze on Tablet	3, 226	0.47	0.706	
	Tweet Recognition	3, 544	1.39	0.245	
	Factual News Recognition	3, 772	0.54	0.657	
	Visual News Recognition	3, 844	1.55	0.200	
Attention PHY	IBI	3, 1448	0.43	0.731	
Media exp. SR	Tweet Liking	3, 527	0.10	0.958	
	Tweet Involvement	3, 509	1.35	0.258	
	Tweet Reading Effort	3, 499	1.66	0.174	
	News Liking	3, 807	1.22	0.301	
	News Involvement	3, 807	0.44	0.727	
	News Comprehension	3, 845	0.38	0.764	
	News Newsworthiness	3, 807	2.04	0.107	
	News Trustworthiness	3, 743	1.23	0.298	
	News Consumption Intent	3, 785	1.36	0.254	

Note. Statistics are from models that included several other factors in addition to mood, as specified in "Data Analysis" section of main article. Only mood condition results are displayed for brevity. SR = self-report; BEH = behavioral; PHY = physiological; SAM = self-assessment manikin; EMG = facial electromyography; ZM = *zygomaticus major* muscle; CS = *corrugator supercilii* muscle; OO = *orbicularis oculi* muscle; IBI = inter-beat interval; iSCR = integrated skin conductance response.

[a]Welch-Sattertwaite approximation (rounded to the closest integer). Note that degrees of freedom for the error term depend on the included random variables.

[b]The model included random slopes for mood across participants.

***$p < 0.001$.

Table S1.2. Mean results (with SEs) for emotional valence related variables by mood.

**Variable**	**Joy**	**Relaxation**	**Fear**	**Depression**
SAM Valence	5.26_{a} (0.11)	5.03_{b} (0.11)	4.89_{bc} (0.11)	4.78_{c} (0.11)
EMG-ZM	1.04_{a} (0.03)	0.96_{b} (0.03)	0.93_{c} (0.03)	0.93_{bc} (0.03)
EMG-CS	1.48_{a} (0.06)	1.55_{b} (0.06)	1.62_{c} (0.06)	1.62_{c} (0.06)
EMG-OO	1.09_{a} (0.03)	0.97_{b} (0.03)	0.93_{b} (0.03)	0.94_{b} (0.03)

Note. Means in each row sharing a common subscript are not statistically different at $p < 0.05$. SAM valence was recorded on a 9-step scale (higher values denote higher pleasantness), facial EMG in ln(μV) units, and iSCR in ln(μS) units. SAM = self-assessment manikin; EMG = facial electromyography; ZM = *zygomaticus major* muscle; CS = *corrugator supercilii* muscle; OO = *orbicularis oculi* muscle; iSRC = integrated skin conductance response.

2.2 Media Experience Self-reports

Results for media experience self-reports were analyzed using similar LMM analyses as for other dependent variables (see section Data Analysis in the main article). Multiple comparison correction was not used because these analyses were considered exploratory. Results from statistical analyses are shown in Table S1.3. For brevity, mean values are shown only for significant effects (Tables S1.4 to S1.6). As expected, *a priori* negative news elicited significantly lower likability ratings than *a priori* positive news (Table S1.4). Negative news were also considered more comprehensible and trustworthy than positive news. These findings give limited evidence for a preferential negativity bias in news videos (see [7]).

Table S1.3. LMM analysis results for media experience variables.

**Variable**	**Effect**	**df**[a]	***F***	***p***	
Tweet Liking	News Valence	1, 530	1.19	0.276	
	Tweet Condition	1, 37[d]	2.80	0.103	
	News Valence × Tweet Condition	1, 530	7.71	0.006	**
Tweet Involvement	News Valence	1, 20[b]	2.11	0.162	
	Tweet Condition	1, 37[d]	2.73	0.107	
	News Valence × Tweet Condition	1, 516	0.69	0.407	
Tweet Reading Effort	News Valence	1, 35[c]	0.75	0.393	
	Tweet Condition	1, 502	0.10	0.752	
	News Valence × Tweet Condition	1, 502	3.60	0.058	
News Liking	News Valence	1, 46[bc]	36.68	< 0.001	***
	Tweet Condition	2, 813	4.54	0.011	*
	News Valence × Tweet Condition	2, 813	3.59	0.028	*
News Involvement	News Valence	1, 27[bc]	1.60	0.217	
	Tweet Condition	2, 811	4.13	0.016	*
	News Valence × Tweet Condition	2, 811	1.33	0.264	
News Comprehension	News Valence	1, 22[b]	6.04	0.022	*
	Tweet Condition	2, 853	3.22	0.040	*
	News Valence × Tweet Condition	2, 853	0.53	0.591	
News Newsworthiness	News Valence	1, 30[bc]	1.20	0.282	
	Tweet Condition	2, 812	0.98	0.377	
	News Valence × Tweet Condition	2, 812	2.43	0.089	
News Trustworthiness	News Valence	1, 27[bc]	5.10	0.032	*
	Tweet Condition	2, 745	1.32	0.269	
	News Valence × Tweet Condition	2, 745	4.62	0.010	*
News Consumption Intent	News Valence	1, 25[bc]	3.34	0.079	
	Tweet Condition	2, 791	3.45	0.032	*
	News Valence × Tweet Condition	2, 791	0.40	0.673	

[a]Welch-Sattertwaite approximation (rounded to the closest integer). Note that degrees of freedom for the error term depend on the included random variables.

[b]The model included random intercepts for news stimuli.

[c]The model included random News Valence slopes for participants.

[d]The model included random Tweet Condition slopes for participants.

***$p < 0.001$. **$p < 0.01$. *$p < 0.05$.

Table S1.4. Mean results (with SEs) for media experience variables by news valence.

Variable	Neg. news	Pos. news
News Liking	3.37 (0.18)	4.64 (0.18)
News Comprehension	5.80 (0.10)	5.64 (0.10)
News Trustworthiness	5.52 (0.16)	5.24 (0.16)

Note. Statistics are shown only for variables with significant effects ($p < 0.05$) in Table S1.3.

News videos presented with negative tweets were considered less likable and involving than news videos presented with positive tweets or without any tweets (Table S1.5). Negative tweets also elicited lower consumption intent ratings than positive tweets. These findings demonstrate that negative tweets elicited not only negative emotions but influenced individuals' evaluations of news videos as well. The findings are consistent with a previous study [8] which demonstrated that twitter messages influence the viewers' evaluations of televised performances. The present investigation extends these findings to media experiences elicited by news broadcasts. Finally, news videos paired with negative tweets also received lower comprehension ratings than news videos without tweets (Table S1.5), which suggests that simultaneous negative tweets made news videos cognitively more demanding.

Table S1.5. Mean results (with SEs) for media experience variables by Tweet condition.

Variable	Neg. tweets	Pos. tweets	No tweets
News Liking	3.85_{a} (0.15)	4.11_{b} (0.15)	4.07_{b} (0.15)
News Involvement	3.79_{a} (0.17)	4.01_{b} (0.17)	4.04_{b} (0.17)
News Comprehension	5.64_{a} (0.10)	5.72_{ab} (0.10)	5.79_{b} (0.10)
News Consumption Intent	1.65_{a} (0.12)	1.82_{b} (0.12)	1.71_{ab} (0.12)

Note. Statistics are shown only for variables with significant effects ($p < 0.05$) in Table S1.3. Means in each row sharing a common subscript are not statistically different at $p < 0.05$.

Significant interaction effects between news valence and tweet condition showed that negative tweets elicit decreased tweet and news likability ratings only when paired with positive news (Table S1.6). This finding is consistent with the principle of negativity dominance [9], which predicted that weak negative stimuli (tweets) would shift evaluations even when paired with proportionally stronger positive stimuli (news videos) but not when paired with similar strong negative stimuli. Table S1.6 also suggests that positive but not negative news were considered more trustworthy when presented without any tweets.

Table S1.6. Mean results (with SEs) for media experience variables by news valence and Tweet condition.

	Negative News			Positive News		
**Variable**	**Negative**	**Positive**	**None**	**Negative**	**Positive**	**None**
Tweet Liking	3.08_a (0.21)	3.03_a (0.21)	-	2.72_a (0.21)	3.18_b (0.21)	-
News Liking	3.36_a (0.19)	3.40_a (0.19)	3.36_a (0.19)	4.33_a (0.19)	4.81_b (0.19)	4.77_b (0.19)
News Trustworthiness	5.58_a (0.17)	5.50_a (0.17)	5.48_a (0.17)	5.10_a (0.17)	5.22_a (0.17)	5.40_b (0.17)

Note. Statistics are shown only for variables with significant effects ($p < 0.05$) in Table S1.3. Means in each row sharing a common subscript (but separately for negative and positive news videos) are not statistically different at $p < 0.05$.

References

1. Derryberry D, Tucker DM. Motivating the focus of attention. In: Neidenthal PM, Kitayama S, editors. The heart's eye: Emotional influences in perception and attention. San Diego, CA: Academic Press; 1994. p. 167–96.
2. Fredrickson BL, Branigan C. Positive emotions broaden the scope of attention and thought-action repertoires. Cogn Emot. 2005;19(3): 313–32. doi: 10.1080/02699930441000238
3. Schwarz N. Feelings-as-information theory. In: Van Lange P, Kruglanski A, Higgins ET, editors. Handbook of theories of social psychology. Thousand Oaks, CA: Sage; 2011. p. 289–308.
4. Ravaja N, Saari T, Kallinen K, Laarni J. The role of mood in the processing of media messages from a small screen: Effects on subjective and physiological responses. Media Psychol. 2006;8(3): 239–65. doi: 10.1207/s1532785xmep0803_3
5. Helle M, Ravaja N, Heikkilä H, Kallenbach J, Kankainen A, Kätsyri J, et al. A theoretical model for media experience and research methods for studying MX . 2011. Available from: "http://virtual.vtt.fi/virtual/nextmedia/Deliverables-2011/D0.4.1_0.4.2_MEDIAEXPERIENCE_A Theoretical Model of Media Experience.pdf"
6. Voorveld HAM. Media multitasking and the effectiveness of combining online and radio advertising. Comput Human Behav. 2011;27(6): 2200–6. Doi: 10.1016/j.chb.2011.06.016
7. Shoemaker PJ. Hardwired for news: Using biological and cultural evolution to explain the surveillance function. J Commun. 1996;46(3): 32–47. doi: 10.1111/j.1460-2466.1996.tb01487.x
8. Cameron J, Geidner N. Something old, something new, something borrowed from something blue: Experiments on dual viewing TV and twitter. J Broadcast Electron Media. 2014;58(3): 400–19. doi: 10.1080/08838151.2014.935852
9. Rozin P, Royzman EB. Negativity bias, negativity dominance, and contagion. Personal Soc Psychol Rev. 2001;5(4): 296–320. doi: 10.1207/S15327957PSPR0504_2
