# Supplementary material for: Negativity Bias in Media Multitasking: The Effects of Negative Social Media Messages on Attention to Television News Broadcasts
Source: PLoS One. 2016 May 4;11(5):e0153712. doi: 10.1371/journal.pone.0153712 (PMC4856346; doi:10.1371/journal.pone.0153712)
Supplement: S2 Appendix — (PDF) [file pone.0153712.s002.pdf]

S2 Appendix: Linear Mixed Model Equations

1 Mixed Models for Self-report and Behavioral Data

Our experimental design for self-report and behavioral data included randomly selected participants who were each exposed to the same randomly selected set of news stimuli, where the news stimuli were assigned into the experimental conditions depending on the counterbalancing condition (i.e., participant group). Ignoring counterbalancing, the design included two crossed random effects, participants and news stimuli, and experimental conditions that varied within participants but between stimuli [1,2]. A conclusive general model for such design can be defined as

$$Y_{ij} = \beta_0 + \beta_1 C_{ij} + u_{0i} + v_{0j} + u_{1i} C_{ij} + e_{ij} \quad (1)$$

where *i* refers to participant, *j* to stimulus, Y is the outcome variable, and C is the experimental condition [1,2]. This model contains a parameter for intercept, β_0, a parameter for the slope of the fixed condition effect, β_1, random intercepts for both participants and stimuli, u_{0i} and v_{0j}, random slope varying across participants for the condition effect, u_{1i}, and the residual error term, e_{ij}. Equation (1) can be restructured as

$$Y_{ij} = (\beta_0 + u_{0i} + v_{0j}) + (\beta_1 + u_{1i}) C_{ij} + e_{ij} \quad (2)$$

Given that each random component is expected to follow a normal distribution with zero mean and unknown variance, equation (2) can be understood intuitively such that the fixed intercept and slope effects (β_0 and β_1) are adjusted by random terms that are allowed to vary between participants (u_{0i} and u_{1i}) or stimuli (v_{0j}) [3].

Equation (2) can be extended to the present experimental design as

$$\begin{aligned} Y_{ij} = {} & (\beta_0 + u_{0i} + v_{0j}) + (\beta_1 + u_{1i}) NESWSVAL_j + (\beta_2 + u_{2i}) TWEETVAL_{ij} \\ & + (\beta_3 + u_{3i}) NESWSVAL_j \times TWEETVAL_{ij} + (\beta_4 + u_{4i}) MOOD_{ij} \\ & + \beta_5 GROUP_i + e_{ij} \end{aligned} \quad (3)$$

where the condition C has been replaced with the present independent variables. Because the valence of specific news videos cannot change, *NEWSVAL* varies only across stimuli *j* (hence subscript $NEWSVAL_j$), whereas *TWEETVAL* and *MOOD* vary both across stimuli *i* and participants *j* ($TWEETVAL_{ij}$ and $MOOD_{ij}$). Participant-level variable *GROUP* is included to account for counterbalancing. Note that equation (3) is simplified for didactic purposes, as the present categorical variables would in reality be coded by $n - 1$ dummy variables and their interactions by $(n - 1) \times (m - 1)$ dummy variables, where *n* and *m* refer to the number of levels in the respective conditions [4]. Because of the complexity of the design, covariances between random terms (e.g., participant intercepts and slopes) are not included in the model. In terms of SPSS syntax, equation (3) can be written as

```
MIXED y BY newsval tweetval mood group
/FIXED = intercept newsval tweetval newsval*tweetval mood group
/RANDOM = intercept newsval tweetval newsval*tweetval mood | SUBJECT(subjid)
/RANDOM = intercept | SUBJECT(newsid)
/METHOD = REML.
```

In practice, *MOOD* variable failed to elicit significant effects for most variables and it was hence included only for the emotional self-report and physiological variables. Following previous guidelines, random intercepts for news stimuli and random slopes for participants were included only when they were estimable (see page 349 in [5]) and at least marginally significant ($p < 0.20$) [1,2,6]. Random intercept parameter for participants was always included in the model.

2 Mixed Models for Physiological Data

Physiological data included time epochs (i.e., varying number of 5-s intervals) and physiological baseline levels as additional variables. Equation (3) was extended to include both of these variables as

$$\begin{aligned} Y_{ijt} = {} & (\beta_0 + u_{0i} + v_{0j}) + (\beta_1 + u_{1i})NESWSVAL_j + (\beta_2 + u_{2i})TWEETVAL_{ij} \\ & + (\beta_3 + u_{3i})NESWSVAL_j \times TWEETVAL_{ij} + (\beta_4 + u_{4i})MOOD_{ij} \\ & + (\beta_5 + u_{5i})EPOCH_{ijt} + \beta_6 GROUP_i + \beta_7 BASELINE_i + e_{ij} \end{aligned} \tag{4}$$

where the new index *t* refers to time. *BASELINE* and *EPOCH* were both coded as continuous variables. Covariance matrix for the residual error term was modified to take the dependency between consecutive measurements into account. Specifically, residual error covariance matrix was defined as

$$R = \sigma^2 \begin{pmatrix} 1 & \rho^1 & \rho^2 & \cdots & \rho^n \\ & 1 & \rho^1 & \cdots & \rho^{n-1} \\ & & 1 & \cdots & \rho^{n-2} \\ & & & \ddots & \vdots \\ & & & & 1 \end{pmatrix} \tag{5}$$

where n refers to the number of epochs, σ^2 to a homogeneous error variance component and ρ^i to correlation between epochs that are i steps apart. In terms of SPSS syntax, equation (4) with error covariance matrix (5) was defined as

```
MIXED y BY newsval tweetval mood group WITH epoch ybaseline
/FIXED = intercept newsval tweetval newsval*tweetval mood group epoch ybaseline
/RANDOM = intercept newsval tweetval newsval*tweetval mood epoch
| SUBJECT(subjid)
/RANDOM = intercept | SUBJECT(newsid)
/REPEATED = epoch | SUBJECT(partid*newsid) COVTYPE(AR1)
/METHOD = REML.
```

As above, *MOOD* failed to exert significant effects for most physiological variables and was only included for emotional self-report and physiological variables. Similarly, random slope and random intercept parameters were included only when they were estimable and at least marginally significant (random intercepts were always included for participants).

References

1. Hoffman L, Rovine MJ. Multilevel models for the experimental psychologist: foundations and illustrative examples. Behav Res Methods. 2007;39(1): 101–17. doi: 10.3758/bf03192848
2. Judd CM, Westfall J, Kenny D a. Treating stimuli as a random factor in social psychology: A new and comprehensive solution to a pervasive but largely ignored problem. J Pers Soc Psychol. 2012;103(1): 54–69. doi: 10.1037/a0028347
3. Baayen RH, Davidson DJ, Bates DM. Mixed-effects modeling with crossed random effects for subjects and items. J Mem Lang. 2008;59(4): 390–412. 10.1016/j.jml.2007.12.005
4. Snijders BAK, Bosker RJ. Multilevel Analysis: An Introduction to Basic and Advanced Multilevel Modeling. London: Sage Publications; 1999.
5. Kenny DA, Kashy DA, Cook WL. Dyadic Data Analysis. New York: The Guilford Press; 2006.
6. Nezlek JB. An Introduction to multilevel modeling for social and personality psychology. Soc Personal Psychol Compass. 2008;2(2): 842–60. doi: 10.1111/j.1751-9004.2007.00059.x
